# Composite Hierarchical Anti-Disturbance Control with Multisensor Fusion for Compact Optoelectronic Platforms

**DOI:** 10.3390/s18103190

**Published:** 2018-09-21

**Authors:** Yutang Wang, Dapeng Tian, Ming Dai

**Affiliations:** 1 Changchun Institute of Optics, Fine Mechanics and Physics, Chinese Academy of Sciences, Changchun 130033, China; ytwang@ciomp.ac.cn (Y.W.); daim@vip.sina.com (M.D.); 2 University of Chinese Academy of Sciences, Beijing 100049, China; 3 Key Laboratory of Airborne Optical Imaging and Measurement, Chinese Academy of Sciences, Changchun 130033, China; 4 Harbin Institute of Technology, Harbin 150001, China

**Keywords:** compact optoelectronic platforms, composite hierarchical anti-disturbance control, adaptive differential evolution algorithm, phase-lag-free multisensor fusion

## Abstract

In the aerospace field, compact optoelectronic platforms (COPs) are being increasingly equipped on unmanned aircraft systems (UAS). They assist UAS in a range of mission-specific tasks such as disaster relief, crop testing, and firefighting. However, the strict constraint of structure space makes COPs subject to multi-source disturbances. The application of a low-cost and low-precision sensor also affects the system control performance. A composite hierarchical anti-disturbance control (CHADC) scheme with multisensor fusion is explored herein to improve the motion performance of COPs in the presence of internal and external disturbances. Composite disturbance modelling combining the characteristic of wire-wound moment is presented in the inner layer. The adaptive mutation differential evolution algorithm is implemented to identify and optimise the model parameters of the system internal disturbance. Inverse model compensation and finite-time nonlinear disturbance observer are then constructed to compensate for multiple disturbances. A non-singular terminal sliding mode controller is constructed to attenuate disturbance in the outer layer. A stability analysis for both the composite disturbance compensator and the closed-loop system is provided using Lyapunov stability arguments. The phase lag-free low-pass filter is implemented to interfuse multiple sensors with different order information and achieve satisfactory noise suppression without phase lag. Experimental results demonstrate that the proposed CHADC strategy with a higher-quality signal has an improved performance for multi-source disturbance compensation.

## 1. Introduction

The continuing rapid development of unmanned aircraft systems (UAS) in the aerospace field has led its essential component, namely optoelectronic platforms, to evolve to a more compact and flexible type. In this way, they can be applied to a growing number of aerospace applications at a more compact size and a lower cost, including aviation planning, forest fire prevention, precision agriculture, disaster relief and public safety, among others [1,2,3,4]. Compact optoelectronic platforms (COPs) serve to isolate various disturbances inside the platform and from the aerospace environment. System nonlinearity, model uncertainties, carrier movement and other internal and external disturbances are significant challenges in these kinds of platforms [5,6,7,8]. Some composite disturbances caused by space minimisation may further deteriorate the system control accuracy and dynamic performance. The composite disturbances affect the control accuracy and dynamic performance and further influence the imaging effect of COPs. Therefore, these disturbances must be compensated for and suppressed.

A wide variety of studies on anti-disturbance have been developed by researchers and practitioners in different industrial sectors to achieve a better disturbance rejection performance [9]. The anti-disturbance methods can be divided into disturbance suppression methods and disturbance compensation methods. The disturbance compensation methods include disturbance observer-based compensation methods and disturbance model-based compensation methods.

The disturbance suppression method aims to design a robust controller such that the system can maintain stability and good performance in the presence of disturbance and model uncertainty. The sliding mode control (SMC) is a robust method of controlling nonlinear and uncertain systems that keeps the systems insensitive and robust to uncertainties and disturbance on the sliding surface [10]. Kinematic and dynamic models of the robot are partly structurally unknown in practice; hence, an adaptive robust control (ARC) of fully constrained cable driven parallel robots is studied, which does not require pre-knowledge of the uncertainties upper bounds and linear regression form [11]. A robust H-infinity output feedback controller is employed to ensure that the dynamic performance of a system with non-holonomic constraints is considered for control design, where the system is subjected to model perturbations and external disturbances [12]. However, the design target of these methods is the convergence of error between command and response, but not targeted compensate for the disturbances. The effects of the disturbance and the system tracking error are considered simultaneously. The control performance is limited when the system disturbance is large.

To achieve complete control of disturbance, the disturbance observer-based control (DOBC) is designed to estimate and compensate for disturbance by filtering the differences between the control input and the ideal input using the inverse model of nominal plant [13]. The robustness and stability of DOBC are analysed, and a reaction torque observer-based robust motion control system is proposed in [14]. The traditional DOBC is designed in frequency domain. To estimate states and disturbance simultaneously, active disturbance rejection control (ADRC) is designed in time domain, in which the disturbance and uncertainty are extended as system state, and a state observer is designed [15]. The nonlinear function in ADRC exhibits a good anti-disturbance performance and can improve the dynamic performance of practical systems [16]. A nonlinear disturbance observer is derived to overcome the disadvantages of some disturbance observers designed or analysed by linear system techniques [17]. To solve the mismatched disturbances and uncertainties of the airbreathing hypersonic vehicle, a nonlinear disturbance observer is employed for fast disturbance estimation and to stabilise the nominal nonlinear dynamics [18]. The equivalent input disturbance estimator-based control (EIDEBC) is employed to estimate an equivalent disturbance on the control input channel and avoid the differentiation of the measured output [19]. An improved EIDEBC approach is presented to increase the flexibility of the system design and apply the proposed approach to a nonminimum-phase plant [20]. The system disturbances can generally be divided into two categories. One is the internal disturbance caused by the internal structure, framework movement and other reasons inside the structure, such as mass unbalance moment, friction, torque fluctuation, wire-wound moment, etc. The other is the external disturbance caused by external factors from the external environment, such as carrier shaking and wind winding. The disturbances, model and parameter uncertainties are considered as an equivalent disturbance in the aforementioned disturbance observer-based compensation methods. The characteristic and the mechanism of the variety of disturbances are ignored. Therefore, this kind of disturbance compensation method is unable to compositely compensate for the disturbance. The upper bound of the equivalent disturbance to be compensated for is relatively large, which brings pressure to the controller design.

Disturbance model-based compensation methods establish the disturbance model according to their characteristics and the mechanism influence on the system performance and compensate for them in the feedforward loop of the controller. Maxwell-slip (GMS) friction-model-based feedforward is applied to acquire sufficiently high path and tracking accuracy [21]. The Stribeck friction model [22,23] and the LuGre model [24,25] have been applied to platforms to compensate for the friction disturbance. These models can well explain the low speed creep or low speed wobble phenomenon of the platforms. In [26], torque ripple is extracted using proper filtering and amplification of the estimated motor speed signal. A new torque ripple minimisation method is also proposed for a switched reluctance motor. In [27], the analysis of the changing rule of the deflecting angle and the arm of force is introduced to compensate for the mass unbalance disturbance. A fuzzy Logic-based disturbance compensator is presented to enhance the tracking performance and contouring accuracy and approximate the unknown non-matching uncertainty of the system [28]. A non-linear disturbance model estimate for a measurable disturbance is adapted for the rejection of the disturbance affecting a closed-loop system via an adaptive neural network compensator [29]. These kinds of methods consider the characteristics of the disturbance; however, compensating for the external disturbances and other unmodeled dynamics is difficult. Therefore, when the system suffers from complex disturbances, the system performance is limited by simply using disturbance model-based compensation methods.

The composite hierarchical anti-disturbance control (CHADC) for complex systems with multiple disturbances has been proposed by Prof. Guo. It combines the respective advantages of disturbance compensation and disturbance suppression. The CHADC approach generally employs two layers: the inner layer, which includes the disturbance observer and the other compensator in the feedforward path, and the outer layer, which includes the disturbance attenuation controller. In the inner layer, multiple disturbances are classified and modelled according to their characteristics and the mechanism influence on the system performance. The disturbances are then targeted, evaluated and compositely compensated for. Meanwhile, the disturbance attenuation method is applied in the outer layer to suppress system equivalent disturbances, such as unmodeled dynamics, parameters and structural uncertainties [30]. The CHADC has been successfully applied in some engineering systems to achieve a relatively better dynamic performance. In [31], the CHADC method combined with a sliding mode controller and a disturbance observer has been presented to a quadrotor UAV in the presence of matched and mismatched disturbances. In [32], to handle the multiple disturbances, the hierarchical control strategy for a magnetically suspended control moment gyro is established, which includes a state-space disturbance observer and a robust H∞ strategy. The cooperation of the hierarchical structure of CHADC guarantees that only a small amount of uncompensated disturbance residual from the inner layer needs to be compensated for in the outer layer, which makes it easier to design the controller in the outer layer. Meanwhile, the tracking performance of the system can be further improved because of the existence of a robust controller in the outer layer.

COPs are widely installed in UAS. To achieve a better imaging effect, COPs have high requirements on both system control accuracy and anti-disturbance performance. For COPs, completing all signal transmissions with conducting rings only is not possible because of the strict constraints of structure space and cost. The signal transmission between shafts is unavoidable through wires. For a more functional platform, more signal must be transmitted through wires; thus, wire-wound moment and other related disturbances are magnified on such kind of platforms. Consequently, for COPs, the identification and modelling of internal disturbance, including wire-wound moment, are urgent and necessary. The strand overall mechanical behaviour is modelled according to the Euler–Bernoulli beam theory to build a link between the structural theories for large-scale analyses of cable structures [33]. Multi-strand wire ropes are physically modelled to predict the global stiffness of the rope in [34]. However, most of these theories have been made to physically characterise the mechanical properties of wire ropes. Only a few focused on the specific impact of high-moment wire-wound disturbance on the accuracy and dynamic performance of COPs and targeted disturbance compensation and control method.

COPs are subject to multiple external and internal disturbances, model and parameter uncertainties in the aerospace environment. Motivated by the idea of the CHADC scheme, this study proposes a composite hierarchical anti-disturbance control strategy for COPs. In the inner layer, it combines a feed-forward inverse model compensation based on parameter identification with the adaptive mutation differential evolution (MDE) algorithm and a finite-time nonlinear disturbance observer to compensate for multiple disturbances. The role of the feed-forward inverse model compensation is to estimate and compensate for the modellable internal disturbance. The finite-time disturbance observer aims to compensate for the effect of the external disturbance in finite time. In the outer layer, a non-singular terminal sliding mode (NTSM) is applied to attenuate disturbance and realise a high dynamic performance. Through composite disturbance estimation and compensation, the NTSM controller may take a smaller value for switching gain without sacrificing the disturbance suppression effect and chattering reduction. This hierarchical structure not only simplifies the design method, but also improves the control performance of the system. In particular, a phase lag-free low-pass filter (phase lag-free LPF) is practically applied to obtain a high-quality signal without phase lag instead of LPF. To interfuse multisensor information, the phase-lead information in the measured signal of a higher-order sensor is used to revise the phase lag in an LPF. Combined with this signal fusion method, the proposed CHADC can more effectively improve the control performance of the system. In addition, the stability of the disturbance compensator and the feedback controller is demonstrated.

This paper is organised as follows: we shall first briefly discuss the motion model of COPs in Section 2; the composite hierarchical anti-disturbance control strategy for COPs and the phase lag-free multisensor fusion are introduced in Section 3; the experiments are performed to verify the effectiveness of the proposed approach, with the results being discussed in Section 4; and finally, the conclusions are presented in Section 5.

## 2. Modelling of the Compact Optoelectronic Platform

**Figure 1:**
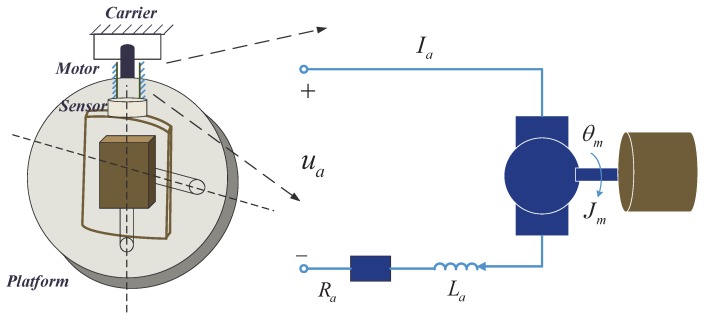
Platform system configuration.

The axes of the COP frame are decoupled, with each degree of freedom being a typical motion control servo system. The outer frame of the compact platform driven by a DC motor was investigated herein. Figure 1 presents the platform system configuration with a motor-driven mechanism-load model. The mathematical model analysis of the platform is as follows:

The motor stator of COPs is embedded in the carrier, indicating that the stator and the carrier are fixed as a rigid body. Meanwhile, the motor rotor is connected with the outer frame through the structure part. The rotor and the frame can be considered as completely rigid when the stiffness of the structural parts is large enough. Carrier shaking disturbance is coupled with the control plant through the motion between the motor stator and the rotor.

The system dynamic equations can be expressed as follows:

(1) $$R_{a}i+L_{a}\frac{di}{dt}+K_{e}{\dot{\theta }}_{m}=u_{a},$$

(2) $$M_{out}=K_{t}i,$$

(3) $$J_{m}{\ddot{\theta }}_{m}+B_{m}{\dot{\theta }}_{m}=M_{out}+M_{di}+M_{de},$$

where $\theta_{m}$ is the rotation angle; $R_{a}$ is the armature resistance; $L_{a}$ is the armature inductance; $u_{a}$ is the armature voltage; Ke is the back-EMF coefficient; $M_{out}$ is the motor output torque; $K_{t}$ is the electromagnetic torque constant; $J_{m}$ is the rotating inertia; $B_{m}$ is the damping coefficient; $M_{di}$ is the internal disturbance moment, including friction moment, wire-wound moment, mass unbalanced moment, unmodeled error, etc.; and $M_{de}$ is the external disturbance moment, including wind disturbance, carrier shaking and other disturbances from the aerospace environment or the UAS motion.

The following equation for $M_{out}$ can be obtained from Equations (1) and (2):

(4) $$M_{out}=\frac{K_{t}}{R_{a}}\left(u_{a}-K_{e}{\dot{\theta }}_{m}-L_{a}\frac{dM_{out}}{dt}\right).$$

Therefore, according to Equations (3) and (4), the electromechanical model of the COPs is:

(5) $$J_{m}{\ddot{\theta }}_{m}+B_{m}{\dot{\theta }}_{m}=\frac{K_{t}}{R_{a}}\left(u_{a}-K_{e}{\dot{\theta }}_{m}-L_{a}\frac{dM_{out}}{dt}\right)+M_{di}+M_{de}.$$

Armature inductance is generally relatively small in COPs. Consider the dynamics induced by armature inductance as part of the internal disturbance, the order of control plant as Equation (5) could be reduced and simplified. Setting $J_{p}=R_{a}J_{m}/K_{t}$, $B_{p}=\left(R_{a}B_{m}+K_{e}K_{t}\right)/K_{t}$, $U=u_{a}$, $M_{inta}=R_{a}M_{di}/K_{t}-L_{a}\frac{dM_{out}}{dt}$, $M_{ext}=R_{a}M_{de}/K_{t}$, $M_{ta}=M_{inta}+M_{ext}$, the differential equation of the platform can then be presented as:

(6) $$J_{p}{\ddot{\theta }}_{m}+B_{p}{\dot{\theta }}_{m}=U+M_{ta},$$

where $J_{p}$ and $B_{p}$ are practical model parameters.

Model and parameter uncertainties $\Delta_{J},\Delta_{B}$ can be found between the system nominal model and the practical model, as shown in (7).

(7) $$\begin{array}{c} J_{p}=\left(1+\Delta_{J}\right)J_{n}\\ B_{p}=\left(1+\Delta_{B}\right)B_{n} \end{array}$$

where $J_{n}$ and $B_{n}$ represent the system nominal inertia and the damping coefficient, respectively. The model and parameter uncertainties can be equivalent to a part of the equivalent internal disturbance. The differential equation of the COP is then rewritten as follows:

(8) $$J_{n}{\ddot{\theta }}_{m}+B_{n}{\dot{\theta }}_{m}=U+M_{t},$$

where $M_{t}=M_{int}+M_{ext}$ and $M_{int}=M_{inta}-\Delta_{J}J_{n}{\ddot{\theta }}_{m}-\Delta_{B}B_{n}{\dot{\theta }}_{m}$ refer to equivalent internal disturbance, including model and parameter uncertainties.

The system motion equation can be expressed as follows:

(9) $$\left\{\begin{array}{cc} {\dot{x}}_{1} & =x_{2}\\ {\dot{x}}_{2} & =-\frac{B_{n}}{J_{n}}x_{2}+\frac{1}{J_{n}}U+\frac{1}{J_{n}}M_{t} \end{array}\right.$$

where $x_{1}=\theta_{m},x_{2}={\dot{\theta }}_{m}$ are the system state variables.

## 3. Control Strategy Design of Composite Hierarchical Anti-Disturbance Control

The CHADC control strategy can be divided into two layers: the inner layer that is the disturbance compensation method and the outer layer that is the disturbance suppression method. Composite disturbance compensators, including inverse model compensation and finite-time disturbance observer, were applied herein to evaluate and compensate for multiple disturbances. NTSM control in the feedback control loop is introduced to attenuate disturbance. The required switching gain of the NTSM is normally larger than the upper bound of the disturbances. The upper bound of the disturbances is not easy to determine; hence, the switching gain may be selected as large enough, which will lead to system chattering. The composite disturbance compensator may reduce the influence of disturbance on the feedback control loop; thus, the switching gain of the NTSM must be larger than the upper bound of the disturbance compensation error only, and the system chatting will be effectively reduced. At the same time, a robust feedback controller may further quickly attenuate disturbance. The hierarchically anti-disturbance structure for multiple disturbances has good anti-disturbance ability.

### 3.1. Composite Disturbance Identification, Modelling and Compensation

A feed-forward inverse model compensation based on parameter identification with an MDE algorithm and a finite-time nonlinear disturbance observer were applied herein to compensate for the multiple disturbances of COPs. The role of the feed-forward inverse model compensation is to estimate and compensate for the modellable internal disturbance. The finite-time disturbance observer aims to compensate for the effect of the external disturbance in finite time.

#### 3.1.1. Internal Disturbance Identification, Modelling and Compensation

Internal disturbances, including friction moment, wire-wound moment, mass unbalanced moment, unmodeled error, etc., result in large residual errors and deteriorate the platform performance. An overly simplistic model cannot accurately represent the disturbance characteristics of the system. Thus, characterizing the behaviours of the internal disturbances in COPs is highly desirable.

**(a) Internal Disturbance Modelling**

A traditional disturbance observer was applied on a COP under a static and relatively stable environment to characterise and evaluate the internal disturbance behaviour. The DOBC, which was proposed by Prof. Ohinishi, estimates the equivalent disturbance and uses it as an offset input signal to compensate for the disturbance.

**Figure 2:**
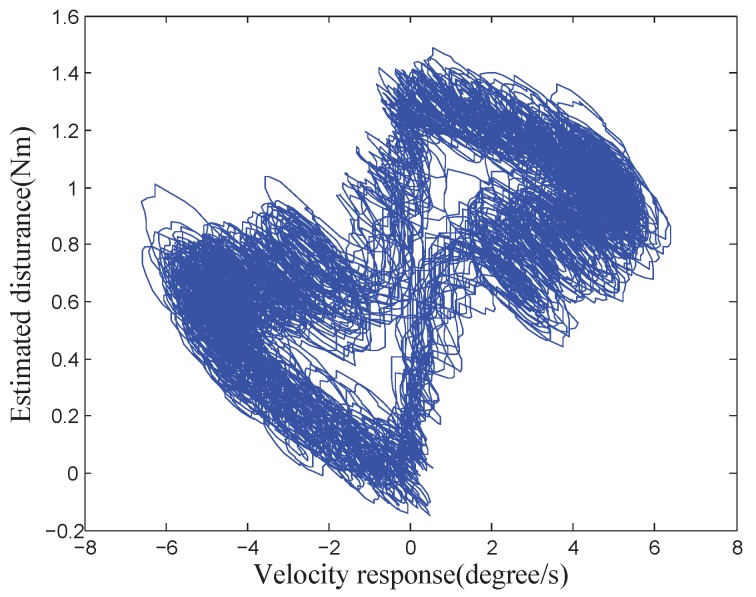
Curve of velocity response and estimated disturbance of a COP.

Figure 2 shows the curve of the velocity response and the estimated disturbance. The disturbance of the platform is complex. Various factors will affect the system performance.

**Figure 3:**
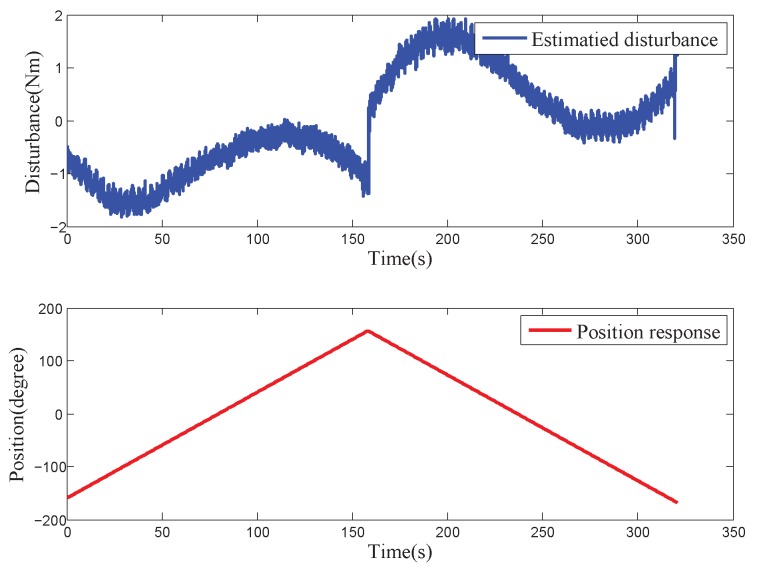
Diagram of triangular position response and estimated disturbance of a COP.

The estimation capability of DOBC is related to the bandwidth of its low-pass filter according to the design principle of the disturbance observer. The disturbance observation result is more accurate when the motion frequency of the system is lower. The COP reciprocates under a 2${}^{\circ }$/s low-speed triangular wave position command in a range of $\pm 150^{\circ }$ to obtain more accurate internal disturbance characteristics in the whole motion area of the platform. Meanwhile, the whole test was conducted in a static and relatively stable environment to avoid the influence of the external disturbance on the system. In this way, the motion of the system is primarily affected by the internal disturbance, which is independent of the external disturbance and the high-frequency attenuation of disturbance observer estimation. Figure 3 depicts the curve of the position response and the estimated disturbance under this circumstance. A certain regular sine/cosine relationship can be observed between the position response and the disturbance.

**Figure 4:**
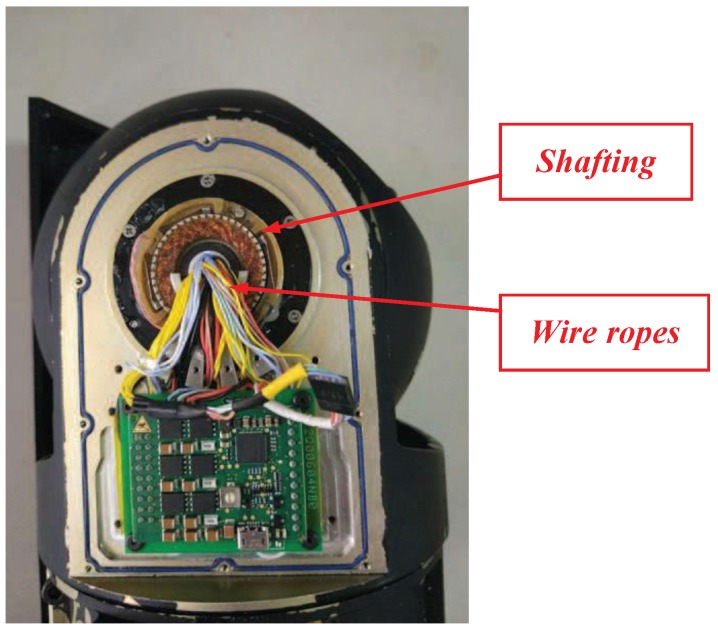
Side elevation drawing of a COP.

The impact of wire-wound friction on the motion control precision for the COP will be exaggerated because a large number of wires must be placed in a restricted space for signal transmission. Figure 4 shows the side elevation drawing of a COP. As can be noted from the figure, a large number of wires must be used for the signal transmission between the shafts. Subjected to tension and torsion of the wire rope strands, the dynamic performance of the platform is directly affected.

The motion of wire rope strands can be simplified as the torsion problem of wire ropes around the equilibrium point. Wire-wound force is generated under the influence of shock excitation when the shaft begins to move with the wire rope strands. The differential equation of the motion can be expressed as follows:

(10) $$J_{w}\ddot{\alpha }+c_{w}\dot{\alpha }+k_{w}\alpha =0,$$

where $J_{w},c_{w},k_{w}$ refer to the wire rope moment of inertia and the damping and stiffness coefficients, respectively. α represents the small angle of the wire rope strands that deviate from the equilibrium position.

Setting $2n_{0}=\frac{c_{w}}{J_{w}},\omega_{w0}=\frac{k_{w}}{J_{w}}$, the characteristic equation of Equation (10) can be converted into:

(11) $$\ddot{\alpha }+2n_{0}\dot{\alpha }+\omega_{w0}^{2}\alpha =0.$$

The general solution of the differential equation in Equation (11) is

(12) $$\alpha =c_{1}e^{\beta_{1}t}+c_{2}e^{\beta_{2}t},$$

where $c_{1}$ and $c_{2}$ are arbitrary constants, $\beta_{1},\beta_{2}=-n_{0}\pm \sqrt{n_{0}^{2}-\omega_{w0}^{2}}$. Three types of motion exist according to the values of $n_{0}$ and $\omega_{w0}$: large damping, small damping and critical damping. Figure 3 shows that the wire-wound motion of the COP belongs to the motion with a small damping, that is, $n_{0}<\omega_{w0}$. Then, $\beta_{1},\beta_{2}=-n_{0}\pm j\sqrt{\omega_{w0}^{2}-n_{0}^{2}}$.

According to Euler’s formula,

(13) $$e^{i\sigma }=cos\sigma +isin\sigma ,$$

Equation (12) can be transformed into

(14) $$\alpha =A_{w}e^{-n_{0}t}sin\left(\sqrt{\omega_{w0}^{2}-n_{0}^{2}}t+\phi_{w}\right),$$

where $A_{w},n_{0},\omega_{w0},\phi_{w}$ is related to the parameters in Equation (10) and the initial state of the wire-wound motion. $A_{w}e^{-n_{0}t}$ can be approximated as a constant *A* when $n_{0}$ is small.

The wire-wound moment $M_{w}$ can be described as follows according to Hooke’s law:

(15) $$M_{w}=k_{w}\alpha .$$

The position angle $x_{1}$ is proportional to time *t* when the system is moving at a constant speed $x_{2}=q_{0}$, then $x_{1}=q_{0}t$ in the zero-initial state. Substituting Equation (15) into (14), the relationship between the disturbance moment and the frame motion angle $x_{1}$ can be obtained as follows:

(16) $$M_{w}=Ak_{w}sin\left(\frac{\sqrt{\omega_{w0}^{2}-n_{0}^{2}}}{q_{0}}x_{1}+\phi_{w}\right).$$

Setting $a_{1}=Ak_{w},a_{2}=\frac{\sqrt{\omega_{w0}^{2}-n_{0}^{2}}}{q_{0}},a_{3}=\phi_{w}$, Equation (16) can be rewritten as follows:

(17) $$M_{w}=a_{1}sin\left(a_{2}x_{1}+a_{3}\right),$$

where $a_{1},a_{2},a_{3}$ are wire-wound parameters to be identified.

Figure 3 shows that the disturbance of the COP can be seen as the sum of the sinusoidal wire-wound moment $M_{w}$ and the Coulomb friction moment $M_{c}$:

(18) $$M_{int}=M_{w}+M_{c},$$

$M_{c}$ is associated with velocity $x_{2}$, which can be described as:

(19) $$M_{c}=\left\{\begin{array}{cc} b_{1}, & if\;x_{2}>0\\ b_{2}, & if\;x_{2}<0\\ 0, & else \end{array}\right.$$

where $b_{1},b_{2}$ are the Coulomb friction parameters to be identified, and they can be obtained from the mean of the estimated disturbance at different velocity directions.

**(b) Disturbance Identification based on Adaptive Mutation Differential Evolution Algorithm (MDE)**

According to Equation (17), $a_{1},a_{2},a_{3}$ are the three parameters to be identified. The classical parameter identification methods include the step response method, frequency response method and least square method, among others. The system identification methods develop all the time as the system becomes more and more complex, and the system demands for a more accurate model. The MDE algorithm is one of the differential evolution(DE) identification algorithms, which is a kind of stochastic optimisation algorithm based on swarm intelligence [35]. The problem of nonlinear identification is converted into an optimisation problem in the parameter space. It is a simple and efficient global optimisation algorithm. The parameter in the early stage can keep individuals diversifying because of the introduction of an adaptive mutation factor, thereby avoiding a premature convergence. The mutation factor is also gradually reduced to obtain the optimal solution.

The basic steps of this algorithm to identify and optimise the parameters in Equation (17) are as follows:

Group Initialization.

If $D_{g}$ parameters are to be identified in the friction model, the expression of the *i*-th individual $X_{gi}$ in the group is shown in Equation (20). In this study, $D_{g}=3$.

(20) $$X_{gi}\left(0\right)=x_{gi}\left(1\right),x_{gi}\left(2\right),\ldots ,x_{gi}\left(D_{g}\right)$$

where $X_{gi}\left(0\right)$ refers to the i-th individual in generation 0. $x_{gi}\left(j\right)$ is a random, uniformly initialised real number in the range $\left[Low_{j},Up_{j}\right]$.

(21) $$X_{gi}\left(j\right)=Low_{j}+rand\left(Up_{j}-Low_{j}\right)$$

where i=1,…,NP, $j=1,\ldots ,D_{g}$, $Low_{j}$ and $Up_{j}$ are the lower and upper bounds of the *j*th parameter range, respectively. NP is the group size. Function rand generates uniformly distributed pseudorandom numbers in the range [0,1].

Differential Mutation.

The vector difference of two random individuals is scaled and combined with the individual vector to be mutated, as in Equation (22).

(22) $$V_{i}\left(t_{g}\right)=X_{gbest}\left(t_{g}\right)+F\left(X_{gp}\left(t_{g}\right)-X_{gk}\left(t_{g}\right)\right)$$

where $t_{g}$ is the generation number; $e_{1}=X_{gp}\left(t_{g}\right)-X_{gk}\left(t_{g}\right)$ is the differential variable; and p≠k. *F* is the mutation factor. An adaptive scaling factor is adopted as follows to avoid a premature algorithm:

(23) $$\lambda =e_{1}^{\left(1-\frac{G_{m}}{G_{m}+1-G}\right)},$$

(24) $$F\left(e_{1}\right)=F_{0}2^{\lambda },$$

where $G_{m}$ is the maximum number of iterations; *G* is the current number of iterations; and $F_{0}$ is the basic mutation factor. $1\leq G\leq G_{m}$, then $1-G\leq (1-\frac{G_{m}}{G_{m}+1-G})\leq 0$. $F=2F_{0}$ when G=1, such that the model parameters in the early stage can keep individuals diversifying to avoid premature convergence. *F* decreases as the number of iteration *G* increases to maintain the optimal solution from destruction. In the iteration process, the violated individual will be randomly regenerated within the boundary range to ensure that the generated mutation vector satisfies the boundary constraint $\left[Low_{j},Up_{j}\right]$ for every individual.

Crossover Operation.

The binomial crossover operation is presented as follows:

(25) $$U_{gi}\left(j\right)\left(t_{g}\right)=\left\{\begin{array}{cc} V_{i}\left(j\right)\left(t_{g}\right) & if\;rand<CR\;or\;j=j_{rand}\\ X_{gi}\left(j\right)\left(t_{g}\right) & otherwise \end{array}\right.$$

where $j_{rand}$ is a random integer between $\left[1,D_{g}\right]$, and CR is a crossover probability.

Competition Operation.

Compared to the new individuals generated by difference variation and cross operation $U_{i}\left(j\right)\left(t_{g}\right)$ with target individual $X_{gi}\left(j\right)\left(t_{g}\right)$ from generation $t_{g}$, the better one goes into the next generation. The competition operation for the minimisation problem is presented as:

(26) $$X_{gi}\left(t_{g}\right)=\left\{\begin{array}{cc} U_{i}\left(t_{g}\right) & if\;f\left(U_{gi}\left(t_{g}\right)\right)<f\left(X_{gi}\left(t_{g}-1\right)\right)\\ X_{gi}\left(t_{g}-1\right) & otherwise \end{array}\right.$$

where f(•) is the adaptive function.

f(•) is chosen herein as:

(27) $$f\left(•\right)={\left(\frac{1}{n_{g}}\overset{n_{g}}{\underset{k=1}{-}}{\left(yout_{w}-M_{w}\right)}^{2}\right)}^{1/2}$$

where $n_{g}$ is the number of samples, and $yout_{w}$ is the measurement disturbance output, $w=1,2,\ldots ,n_{g}$. The decision criteria are the minimum value of the root-mean-square error (RMS) between the measurement output and the estimated output from the optimisation identified model with the measurement input. Thus, the identification problem is transformed into the optimisation problem of the parameter space.

**(c) Inverse Model Compensation**

According to the abovementioned analysis, the internal disturbance is associated with frame motion states $x_{1},x_{2}$. For a closed-loop control system, the error between the control commands and the system response is little if the robust feedback controller in the outer layer works. Therefore, the system performance can be improved by modelling the internal disturbance using position and velocity commands instead of motion response and applying the inverse model in a feedforward loop to compensate for the internal disturbance. A careful design strategy is essential in a feedback approach to avoid instability. Meanwhile, the effectiveness of the feedforward compensator depends on the accuracy of the applied inverse model. The design goal of the inverse model compensation is as follows:

(28) $$u_{ci}=-M_{int},$$

where $u_{ci}$ refers to the control output of the inverse model compensation.

#### 3.1.2. Finite-Time Disturbance Observer

For a COP, the ideal motion equation of the controlled frame without disturbance can be expressed as:

(29) $$\left\{\begin{array}{cc} {\dot{x}}_{n1} & =x_{n2}\\ {\dot{x}}_{n2} & =-\frac{B_{n}}{J_{n}}x_{n2}+\frac{1}{J_{n}}u_{s} \end{array}\right.$$

where $x_{n1},x_{n2}$ are the ideal system position response and the velocity response, respectively. $u_{s}$ is the control output of the feedback controller NTSM.

The error between the expected position response $x_{n1}$ and the real position response $x_{1}$ is defined as follows:

(30) $$e=x_{n1}-x_{1}.$$

According to the design principle of disturbance observer(DOB) in the RIC structure [36],

(31) $$u_{RIC}=gJ_{n}\dot{e}+gB_{n}e.$$

where $u_{RIC}$ is the estimated disturbance, and *g* is the equivalent filter bandwidth of the disturbance observer.

However, a linear filter cannot effectively compensate for the nonlinearity, and the finite-time convergence of error cannot be guaranteed. A nonlinear element is introduced into Equation (31) herein:

(32) $$F_{n}\left(e,\alpha \right)={\left|e\right|}^{\alpha }sgn\left(e\right),0<\alpha <1,$$

where sgn(•) is the standard signum function.

$F_{n}\left(e,\alpha \right)$ is a nonlinear function that varies with the error between the expected response and the practical response. The key point lies in properly designing $F_{n}\left(e,\alpha \right)$ such that the disturbance observer with the finite-time characteristic obtains a faster rate of convergence and a smaller phase lag. It possesses a nonlinear merit, where its gain is substantial compared to the small error, and because of which, the control error converges fast. However, its gain is small for big errors, and because of which, the DA converter will not be saturated in practical applications. The nonlinear characteristic of $F_{n}\left(e,\alpha \right)$ enhances the dynamic performance of the controller and speeds up the system convergence.

Define $u_{fn}$ as the output of the nonlinear function $F_{n}\left(e,\alpha \right)$. The finite-time disturbance observer based on the RIC structure can then be redesigned as follows:

(33) $$u_{cd}=gJ_{n}{\dot{u}}_{fn}+gB_{n}u_{fn},$$

where $u_{cd}$ is the output of the finite-time disturbance observer and refers to the evaluated disturbance by the finite-time disturbance observer.

The control output with disturbance compensation can be expressed as follows:

(34) $$\begin{array}{c} U=u_{s}+u_{ci}+u_{cd}, \end{array}$$

The feedforward loop does not affect the system stability; hence, the stability analysis of the composite disturbance compensation method is equivalent to the analysis of its feedback loop.

Considering only the feedback loop of the disturbance compensation, the system motion equation can be expressed as follows:

(35) $$\left\{\begin{array}{cc} {\dot{x}}_{1} & =x_{2}\\ {\dot{x}}_{2} & =-\frac{B_{n}}{J_{n}}x_{2}+\frac{1}{J_{n}}\left(u_{cd}+M_{t}\right) \end{array}\right.$$

The control output of the finite-time disturbance observer $u_{cd}$ is defined as Equation (33). Define the input of the control plant, including disturbance, as $U^{*}$, then $U^{*}=u_{cd}+M_{t}$.

Define a deformation disturbance $M_{def}$ as the deformation of $M_{t}$, and $M_{t}=gJ_{n}{\dot{M}}_{def}+gB_{n}M_{def}$. Note that if $M_{t}$ is bounded, then $M_{def}$ is also a bounded disturbance, and defined as $|M_{def}|\leq d^{*}g$.

Furthermore, since $u_{cd}$ can be expressed as Equation (33); hence, the effect of disturbance $M_{t}$ on the control plant can be considered as the effect of disturbance $M_{def}$ on the disturbance compensation loop. In this manner, the feedback loop can be transformed, and the transformed system motion equation can be expressed as follows:

(36) $$\left\{\begin{array}{cc} {\dot{x}}_{1} & =x_{2}\\ {\dot{x}}_{2} & =-\frac{B_{n}}{J_{n}}x_{2}+\frac{1}{J_{n}}u_{cd}^{*}, \end{array}\right.$$

The new output of the finite-time disturbance observer $u_{cd}^{*}$ can be written as follows:

(37) $$u_{cd}^{*}=gJ_{n}\left({\dot{u}}_{fn}+{\dot{M}}_{def}\right)+gB_{n}\left(u_{fn}+M_{def}\right).$$

The input of the control plant, including disturbance $U_{c}^{*}$, is:

(38) $$\begin{array}{cc} U_{c}^{*} & =u_{cd}^{*}\\ & =\left(gJ_{n}{\dot{u}}_{fn}+gB_{n}u_{fn}\right)+\left(gJ_{n}{\dot{M}}_{def}+gB_{n}M_{def}\right)\\ & =u_{cd}+M_{t}\\ & =U^{*}. \end{array}$$

According to the above-mentioned analysis, the system motion equation in Equation (35) is equivalent to the system motion equation in Equation (36). Therefore, the stability of the compensation system could be equivalently proven by discussing the stability of the closed-loop system in Equation (36).

Define an intermediate variable $x_{temp}=u_{fn}+M_{def}$. Equation (37) could then be rewritten as follows:

(39) $$u_{cd}^{*}=gJ_{n}{\dot{x}}_{temp}+gB_{n}x_{temp}.$$

Based on Equations (36) and (39), the equivalent relationship can then be obtained as follows:

(40) $$\begin{array}{cc} x_{2} & =gx_{temp}\\ & =gu_{fn}+gM_{def}. \end{array}$$

Based on Equations (30), $e=x_{n1}-x_{1}$. *e* refers to the error between the expected output angular $x_{n1}$ and the practical output angular $x_{1}$. $\dot{e}={\dot{x}}_{n1}-{\dot{x}}_{1}={\dot{x}}_{n1}-x_{2}$. According to Equations (32) and (40), the error *e* based equivalent equation could then be written as follows:

(41) $$\dot{e}=-g{\left|e\right|}^{\alpha }sgn\left(e\right)-M_{eq}+{\dot{x}}_{n1},$$

where $M_{eq}=M_{def}/g$ is the equivalent disturbance and $|M_{eq}|\leq d^{*}$, ${\dot{x}}_{n1}$ is expected system angular velocity. When the system is expected to be stationary, its expected angular velocity ${\dot{x}}_{n1}$ is zero. When the expected velocity ${\dot{x}}_{n1}\neq 0$, it is assumed to be bounded, and $|{\dot{x}}_{n1}|\leq X$.

The error between the expected output and the practical output is caused by multiple disturbances including external disturbances, internal disturbances, model and structure uncertainties. When the multiple disturbances are evaluated and compensated, the error *e* will converge to be zero, and then the practical output will track expected output. It means that the estimated and compensated disturbance by the proposed finite-time disturbance observer will approach practical disturbance. Therefore, the finite-time convergence of the proposed disturbance observer can be proved by the error convergence of the equivalent closed-loop system.

The definitions and the theorems are presented as follows to prove the finite-time stability of the system [37]:

**Definition** **1.** *(Finite-time stability) Considering the system*

(42) $$\dot{x}=f\left(x\right),x\in U_{a}\subseteq R^{n},f\left(0\right)=0,$$

*where $f:U_{a}\to R^{n}$ is a continuous function of the open region of $U_{a}$, and the open area $U_{a}$ contains the origin point. The solution of the system x=0 is finite-time stability if and only if the system is stable and converges in finite time. The finite-time convergence is $\forall x_{0}\in U_{0}\subset R^{n}$. If a continuous function $T\left(x\right):U_{0}\to \left(0,+\infty \right)$ exists, the solution of Equation (42) satisfies the following: when $t\in [0,T\left(x_{0}\right)]$, $x\left(t,x_{0}\right)\in U_{0}$ and $\lim_{t\to T(x_{0})}x\left(t,x_{0}\right)=0$. $x(t,x_{0})=0$ when $t>T(x_{0})$.*

The finite-time stability requires not only the stability of the system, but also the finite-time convergence. The Lyapunov stability criterion for finite-time control systems is:

**Theorem** **1.** *Considering the system in Definition 1, suppose that a continuous differentiable function V:U→R exists and satisfies the following conditions:*

*(1)*  *V is a positive definite function;*
*(2)*  *the arithmetic number c and a satisfy c>0 and a∈(0,1), respectively. The open neighbourhood containing the origin $U_{0}$ satisfies $U_{0}\subseteq U$. If the following condition is established,*
  (43) $$\dot{V}\left(x\right)+cV^{a}\left(x\right)\leq 0,x\in U_{0}$$
  *then, the system shown in Equation (42) is finite-time stable. If $U=U_{0}=R$, and V(x) is radial unbounded, then the system is globally finite-time stable. In addition, the convergence time T satisfies $T\leq \frac{V{\left(x\left(0\right)\right)}^{1-a}}{c(1-a)}$.*

The Lyapunov function is defined as follows:

(44) $$V=\frac{1}{2}e^{2}$$

Substituting Equation (44) into Equation (43), we obtain:

(45) $$\dot{V}+cV^{a}=e\dot{e}+c{\left(\frac{1}{2}e^{2}\right)}^{a}$$

Let a=(1+α)/2,a∈(0,1), then

(46) $$\begin{array}{cc} \dot{V}+cV^{a} & =e\dot{e}+c{\left(\frac{1}{2}e^{2}\right)}^{a}\\ & ={e(-g|e|}^{\alpha }sgn\left(e\right)-M_{eq}+{\dot{x}}_{n1})+c{\left(\frac{1}{2}\right)}^{\frac{1+\alpha }{2}}e^{1+\alpha }\\ & \leq -{g|e|}^{1+\alpha }+\left|e|\right|M_{eq}\left|+|e|\right|{\dot{x}}_{n1}|+c{\left(\frac{1}{2}\right)}^{\frac{1+\alpha }{2}}{\left|e\right|}^{1+\alpha }\\ & \leq -{g|e|}^{1+\alpha }+{\left|e\right|}^{1+\alpha }d^{*}+{\left|e\right|}^{1+\alpha }X+c{\left(\frac{1}{2}\right)}^{\frac{1+\alpha }{2}}{\left|e\right|}^{1+\alpha }\\ & =-{\left|e\right|}^{1+\alpha }\left(g-d^{*}-X-c{\left(\frac{1}{2}\right)}^{\frac{1+\alpha }{2}}\right) \end{array}$$

Therefore, if $g\geq d^{*}+X+c{\left(\frac{1}{2}\right)}^{\frac{1+\alpha }{2}}$, then

(47) $$\dot{V}+cV^{a}\leq 0$$

According to Theorem 1, as long as the conditions in Equations (48) and (49) are guaranteed, the system in Equation (41) is global finite-time stable. The system control error will converge to zero in finite time, and converge time satisfies $T\leq \frac{V{\left(e\left(0\right)\right)}^{1-a}}{c(1-a)}$.

(48) a=(1+α)/2

(49) $$g>d^{*}+X+c{\left(\frac{1}{2}\right)}^{\frac{1+\alpha }{2}}$$

The proposed method also has the following characteristics in the problem of anti-disturbance ability:

**Theorem** **2.** *Let $c_{1}$ be an arbitrarily small constant. The error between the expected output and practical output in Equation (41) will be stabilised into a region Q in finite time, where*

$$Q=e:|e|\leq {\left(\frac{d^{*}+X+c_{1}}{g}\right)}^{1/\alpha }$$

**Proof.**  Select a continuous differentiable Lyapunov function as in Equation (44). The following equation after differentiation can be obtained:

(50) $$\begin{array}{cc} \dot{V}\left(x\right)=e\dot{e} & =-{g|e|}^{1+\alpha }-eM_{eq}+e{\dot{x}}_{n1}\\ & \leq -|e|(g|e{|}^{\alpha }-d^{*}-X) \end{array}$$

The final convergence domain of the proposed controller is defined as:

(51) $$\Omega \propto {\left(\frac{d^{*}+X+c_{1}}{g}\right)}^{1/\alpha },0<\alpha <1,\forall c_{1}>0.$$

For arbitrary e∈R−Q, $|e|>{\left(\frac{d^{*}+X+c_{1}}{g}\right)}^{\left(1/\alpha \right)}$. According to Equation (50), for arbitrary e∈R−Q,

(52) $$\dot{V}\leq -c_{1}\left|e\right|<0.$$

 □

The analysis above proved that the error between the expected output and practical output of the equivalent closed-loop structure will converge to the stabilised region *Q* in finite time. The practical control output will track the expected output and then the estimated disturbance by the proposed finite-time disturbance observer will approach practical disturbance. The finite-time convergence of the proposed disturbance observer is proved.

### 3.2. Phase Lag-Free Sensor Filter

The position or velocity signal is employed as a feedback signal to realise the closed-loop control. The performance of the closed-loop control system depends on the sensor signal-to-noise ratio (SNR). The COP is restricted by the installation space and cost, and the sensor measurement accuracy is limited. For the low SNR sensor, LPFs are commonly used to improve its SNR. However, the associated phase lag will degrade the performance of the whole system.

A traditional LPF is widely used as:

(53) $$\left\{\begin{array}{cc} {\dot{x}}_{f1} & =-g_{f}x_{f1}+g_{f}x_{1}\\ y_{f} & =x_{f1}. \end{array}\right.$$

where $x_{lpf}$ is the filtered state; $y_{f}$ is the filter output; and $g_{f}$ determines the cut-off frequency of the filter.

However, its phase response in Equation (54) shows that the phase lag problem will be introduced by Equation (53). The higher the signal frequency ω, the more obvious the signal phase lag after filtering.

(54) $$\varphi \left(\omega \right)=∠L\left(j\omega \right)=-arctan\left(\frac{\omega }{g_{f}}\right)$$

The phase lag of the filtered signal is unavoidable compared to the original signal because of the existence of a first-order inertial element in the LPF. A higher-order sensor could be used to obtain “phase lead” information.

The phase lag-free LPF [38] with a higher-order sensor can achieve satisfactory noise suppression without a significant phase lag, as in Equation (55). For the low-cost COP, the realisation of motion control with a higher performance can be guaranteed by multisensor fusion. This method is established based on pole-zero cancellation, which has a significant meaning in physics.

(55) $$\left\{\begin{array}{cc} {\dot{x}}_{f1} & =-gx_{f1}+gx_{1}\\ {\dot{x}}_{f2} & =-gx_{f2}+{\dot{x}}_{1}\\ y_{f} & =x_{f1}+x_{f2}. \end{array}\right.$$

where $x_{1f},x_{2f}$ are the filter states of the phase lag-free LPF.

**Figure 5:**
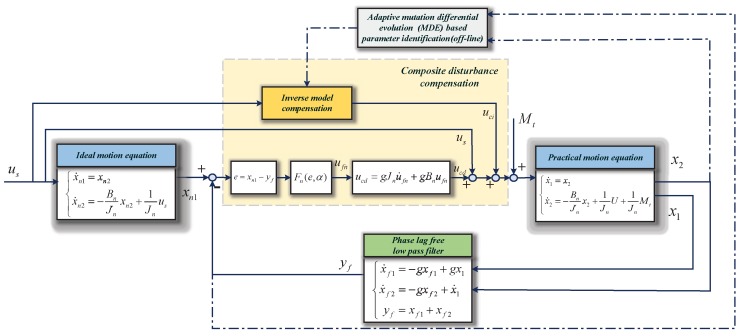
Control strategy of the composite disturbance compensation method in the inner layer.

Figure 5 shows the composite disturbance compensation method in the inner layer of the proposed control strategy CHADC with the phase lag-free LPF.

### 3.3. Disturbance Suppression Control Strategy Based on Non-Singular Terminal Sliding Mode

After the disturbance compensation in the inner layer, the equivalent control plant of the outer controller is presented as follows:

(56) $$\left\{\begin{array}{cc} {\dot{x}}_{1} & =x_{2}\\ {\dot{x}}_{2} & =-\frac{B_{n}}{J_{n}}x_{2}+\frac{1}{J_{n}}u_{s}+\frac{1}{J_{n}}M_{s} \end{array}\right.$$

where $M_{s}$ refers to the disturbance residual of the disturbance compensation method in the inner layer.

The new state variables of the position error and its derivative are defined as follows: $x_{e1}=x_{c}-x_{1},x_{e2}={\dot{x}}_{e1}$, where $x_{c}$ is the expected position command. The control target of NTSM herein is to design the NTSM controller $u_{s}$, such that the position error $x_{e1}\to 0$.

The state space of the outer layer can be expressed as:

(57) $$\left\{\begin{array}{cc} {\dot{x}}_{e1} & =x_{e2}\\ {\dot{x}}_{e2} & ={\ddot{x}}_{c}+\frac{B_{n}}{J_{n}}x_{2}-\frac{1}{J_{n}}u_{s}-\frac{1}{J_{n}}M_{s} \end{array}\right.$$

A non-singular terminal sliding mode method(NTSM) is designed to achieve good performances, such as fast convergence, better tracking precision and robustness to disturbance. The sliding surface [39] is designed as follows:

(58) $$s=x_{e1}+\frac{1}{\beta }x_{e2}^{p/q},$$

where β>0, *p* and *q* are the positive odd integers and 1<p/q<2.

The terminal sliding mode controller can be designed as:

(59) $$u_{s}=J_{n}\left({\ddot{x}}_{c}+\frac{B_{n}}{J_{n}}x_{2}+lsgn\left(s\right)-\beta \frac{q}{p}x_{e2}^{2-p/q}\right).$$

**Assumption** **1.** *The disturbance $M_{s}$ is bounded, and a constant k>0 satisfying $0<|\frac{M_{s}}{J_{n}}|\leq k$ exists.*

We can then reach the following theorem:

**Theorem** **3.** *If Assumption 1 holds, under the control law (59), the control error of COPs converges to zero in finite time if the switching gain satisfies l>k.*

**Proof.**  Choosing Lyapunov function $V=\frac{1}{2}s^{2}$ and taking the derivative of it along with Equation (57) yield:

(60) $$\begin{array}{cc} \dot{V} & =s\dot{s}=s\left(x_{e2}+\frac{1}{\beta }p/qx_{e2}^{p/q-1}{\dot{x}}_{e2}\right)\\ & =s(x_{e2}+\frac{1}{\beta }p/qx_{e2}^{p/q-1}\left({\ddot{x}}_{c}+\frac{B_{n}}{J_{n}}x_{2}-\frac{1}{J_{n}}u_{s}-\frac{1}{J_{n}}M_{s}\right))\\ & =s(x_{e2}+\frac{1}{\beta }p/qx_{e2}^{p/q-1}\left({\ddot{x}}_{c}+\frac{B_{n}}{J_{n}}x_{2}-\frac{1}{J_{n}}J_{n}\left({\ddot{x}}_{c}+\frac{B_{n}}{J_{n}}x_{2}+lsgn\left(s\right)-\beta \frac{q}{p}x_{e2}^{2-p/q}\right)-\frac{1}{J_{n}}M_{s}\right))\\ & =s(-\frac{1}{\beta }p/qx_{e2}^{p/q-1}\left(lsgn\left(s\right)+\frac{1}{J_{n}}M_{s}\right))\\ & \leq -\frac{1}{\beta }p/qx_{e2}^{p/q-1}l|s|+\frac{1}{\beta }p/qx_{e2}^{p/q-1}|\frac{M_{s}}{J_{n}}\left||s\right|\\ & =-\frac{1}{\beta }p/qx_{e2}^{p/q-1}\left|s|(l-\right|\frac{M_{s}}{J_{n}}\left|\right) \end{array}$$

1<p/q<2,β>0,p,q are positive odd numbers; hence, $p/qx_{e2}^{p/q-1}>0$, and $|\frac{M_{s}}{J_{n}}|\leq k<l$. Therefore, it has $\dot{V}\leq 0$ for s≠0.

The existence of the sliding mode is guaranteed from the abovementioned analysis. The states reach the terminal sliding manifold s=0 from any initial condition in finite time.

When s=0, Equation (58) could be transformed into

(61) $$x_{e1}+\frac{1}{\beta }x_{e2}^{p/q}=0.$$

Considering ${\dot{x}}_{e1}=x_{e2}$, Equation (61) could be written as follows:

(62) $$x_{e1}+\frac{1}{\beta }{\dot{x}}_{e1}^{p/q}=0.$$

 □

Equation (62) is a fractional differential equation. The analytic solutions of many fractional differential equations are known to be expressed by some special functions, and solving the analytic solutions of some other fractional differential equations is impossible. However, discussing the convergence of the state $x_{e1}$ in Equation (62) is possible by evaluating the convergence time of the fractional differential equation. If the convergence time can be obtained, the state in the fractional differential equation could converge to zero in finite time.

Equation (62) could be transformed as $x_{e1}^{-q/p}{\dot{x}}_{e1}=-\beta^{q/p}$. Supposing that $t_{ini}$ is the time costed from s(0)≠0 to $s(t_{ini})=0$, the convergence time from s(0)≠0 to $x_{e1}\left(t_{final}\right)=0$ can be described as Equation (63).

(63) $$t_{final}=t_{ini}+\frac{p}{\beta^{q/p}\left(p-q\right)}{\left|x_{e1}\left(t_{ini}\right)\right|}^{1-q/p}.$$

Therefore, the position error will converge to zero along the sliding surface *s* in finite time $t_{final}$. The control target of the NTSM can be achieved.

## 4. Experimental Results and Discussion

### 4.1. Implementation of the Experimental System

**Figure 6:**
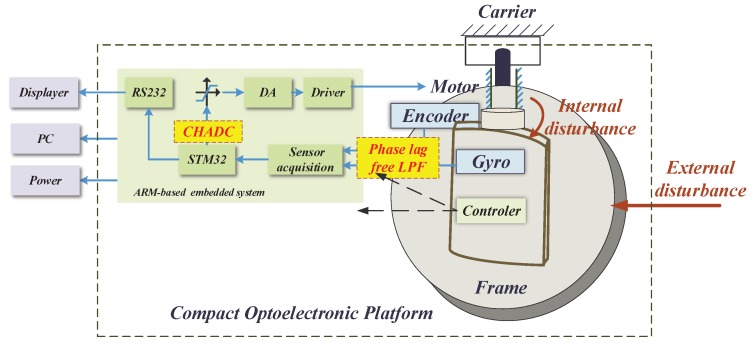
Experimental configuration.

**Figure 7:**
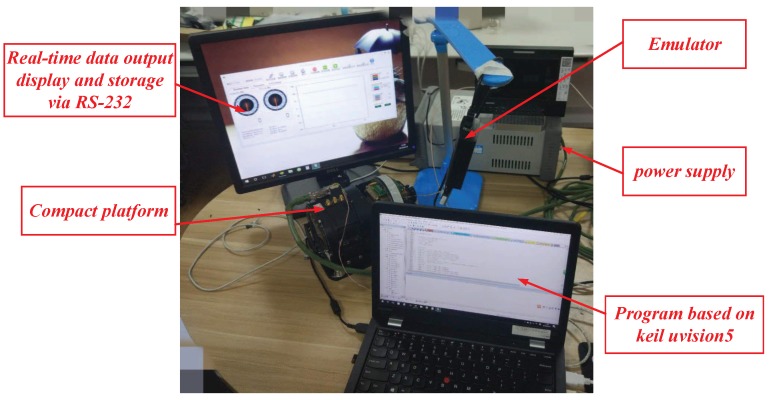
Experimental setup.

Practical experiments were implemented to verify the performance of the proposed system. Figure 6 illustrates the composition of the experimental system, while Figure 7 shows a photograph of the experimental devices. The compact platform was composed of a DC motor, a two-axis encoder, a three-axis gyroscope, motor drivers, sensor acquisition, control circuit, etc. The inner and outer frames were orthogonal to each other in the structural design; hence, the motion coupling between them was small enough be ignored. The inner frame of the two-axis platform was fixed, and the outer frame was taken as an experimental subject herein. In the experiments, the algorithms were realised by programming in an ARM-based (STM32F407) embedded system. The sampling time was 1 ms. All programs were scheduled in C language. Table 1 lists the other parameters.

### 4.2. Experimental Results and Discussions

#### 4.2.1. Sensor Data Processing

The position signal was noisy because of cost constraints and signal interference. Two sets of experiments were performed to verify the effectiveness of the phase lag-free LPF.

**Figure 8:**
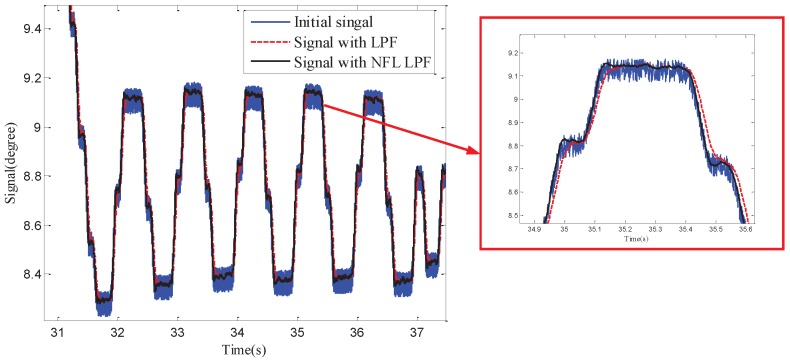
Comparison of initial and filtered signals.

First, shake the COP and make it do sinusoidal motions with different frequencies and amplitude values. Acquire and compare the original position signal, LFP filtering signals and phase lag-free filtering signal (Figure 8). Compared with the LPF algorithm, the phase lag-free LPF algorithm had less influence on the signal phase while simultaneously improving the signal SNR. It is more beneficial in achieving high performance control and disturbance suppression of the control system.

**Figure 9:**
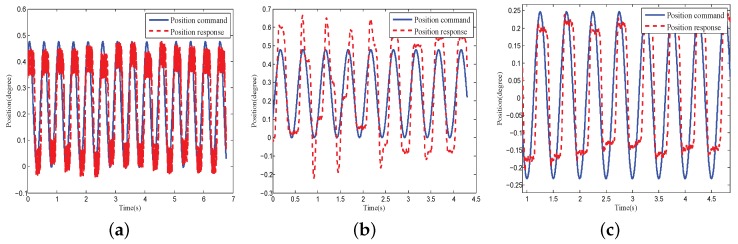
Closed loop performance comparison of initial and filtered signals: (**a**) With initial signal; (**b**) With traditional LPF singal; (**c**) With phase lag-free LPF signal.

In the second experiment, the PID feedback control was applied to the COP. The experiment consisted of three cases. In case 1, the position signal was the initial signal from the position sensor. In case 2, the position signal was filtered by the LPF. In case 3, the position signal was filtered by the phase lag-free LPF with high-order sensor information. Figure 9 shows the comparison results. The effect of the signal phase lag on control precision was more significant when the system control bandwidth was low. Figure 9 shows an obvious control overshoot when LPF was used. This was caused by the additional phase lag in the controlled plant. A better control performance was achieved when a phase lag-free LPF was applied. In all subsequent experiments, the feedback data were filtered through the phase lag-free LPF.

#### 4.2.2. Internal Disturbance Model Identification

**Figure 10:**
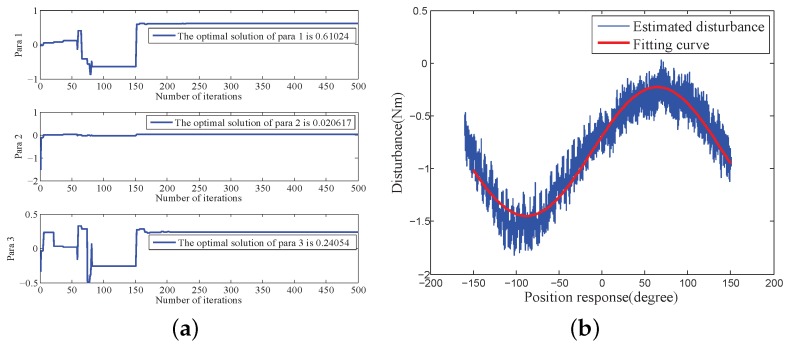
Parameter identification and optimization when velocity > 0: (**a**) Parameter convergence; (**b**) Optimal fitting.

**Figure 11:**
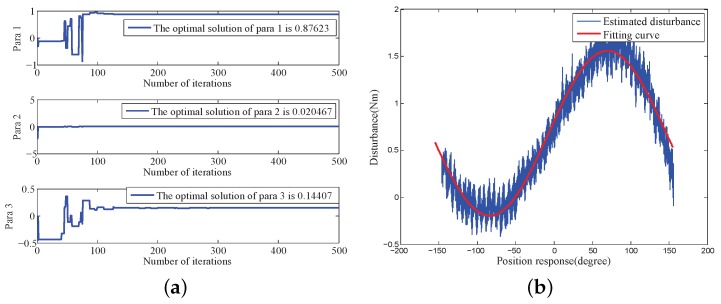
Parameter identification and optimization when velocity <0: (**a**) Parameter convergence; (**b**) Optimal fitting.

According to the test data shown in Figure 3, three parameters in Equation (13) were identified and optimised based on the platform of MATLAB R2012b. Table 2 shows the parameters of the MDE algorithm. Figure 10 and Figure 11 the parameter convergence processes and the optimal parameter fitting results when the velocity of the COP was greater than 0 or less than 0, respectively. The convergent speed of the parameter identification process based on the MDE algorithm was fast. The three nonlinear parameters can be identified within 150 generations. The identification internal disturbance model is presented in Equation (64). The model can be applied for inverse model compensation.

(64) $$M_{int}=\left\{\begin{array}{cc} 0.61024sin(0.020617x_{1}+0.24054)-0.8376 & {\dot{x}}_{1}>0\\ 0.87623sin(0.020467x_{1}+0.13307)+0.6827 & {\dot{x}}_{1}<0\\ 0 & \mathrm{else} \end{array}\right.$$

#### 4.2.3. Finite-Time Disturbance Observer

**Figure 12:**
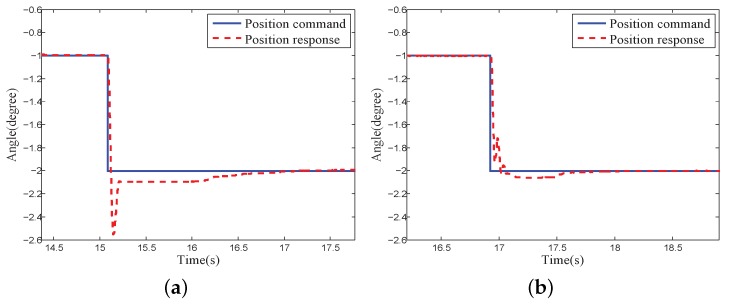
Step response comparison: (**a**) Without finite-time disturbance observer; (**b**) With finite-time disturbance observer.

Figure 12 demonstrates the system step response of the platform with and without the proposal finite-time disturbance observer. The PID controller with the proposal finite-time disturbance observer provided a faster response convergence rate and a smaller overshoot. The convergence time during the position response converging to −2±0.01 was reduced from 1.89 s to 0.91 s, and the overshoot was reduced from 0.55 degree to 0.062 degree.

#### 4.2.4. Disturbance Compensation Performance under Multiple Disturbances

**Figure 13:**
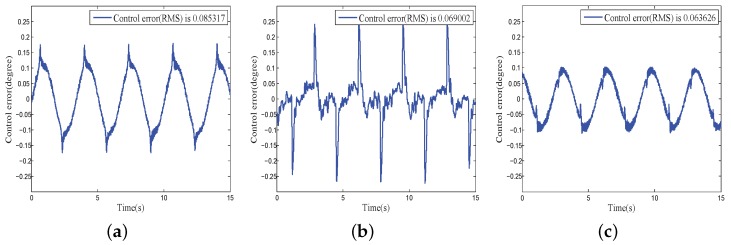
Control error of different disturbance compensation method under internal disturbance: (**a**) finite-time DOB; (**b**) inverse model compensation; (**c**) proposed composite disturbance compensator.

Figure 13 illustrates the experimental control error of the system compensators under internal disturbance with finite-time DOB, inverse model compensation controller and the proposed composite disturbance compensator with inverse model compensation and finite-time DOB. A PID controller with the same parameters was applied in the feedback loop. According to these results, all controllers effectively compensated for the internal disturbance. The control error of the proposed composite disturbance compensator was the smallest.

**Figure 14:**
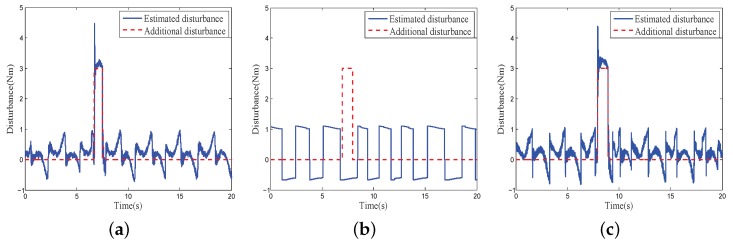
Disturbance Estimation of different disturbance compensation method under external and internal disturbance: (**a**) finite-time DOB; (**b**) inverse model compensation; (**c**) proposed composite disturbance compensator.

**Figure 15:**
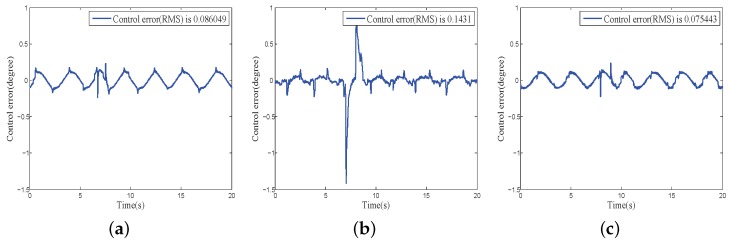
Control error of different disturbance compensation method external and under internal disturbance: (**a**) finite-time DOB; (**b**) inverse model compensator; (**c**) proposed composite disturbance compensator.

Furthermore, when working in a practical environment, the COP also suffers from external disturbance, including wind disturbance and carrier shaking. An additional simulated wind disturbance was imposed on the system to investigate the comparative performance of different compensators with multiple disturbances. Figure 14 and Figure 15 show the experimental results, where a single inverse model compensation controller cannot compensate for the external disturbance, and the proposed composite disturbance compensator can achieve the fastest disturbance evaluation and the lowest control error compared with the other two methods.

The abovementioned experimental results indicated that the proposed composite disturbance compensator evidently had obvious advantages. The proposed method can effectively evaluate and compensate for the influence of the internal and external disturbances on COPs in a finite time.

#### 4.2.5. Performance of the Proposed CHADC for COPs

A set of contrast experiments between the traditional PID feedback controller with the composite disturbance compensation method and the proposed CHADC algorithm was implemented to validate the proposed composite hierarchical anti-disturbance control method on COPs.

**Figure 16:**
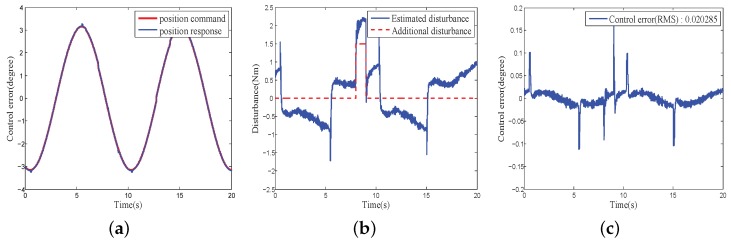
Performance of PID feedback control with proposed disturbance compensation method: (**a**) Control command and response; (**b**) Disturbance estimation; (**c**) Control error.

**Figure 17:**
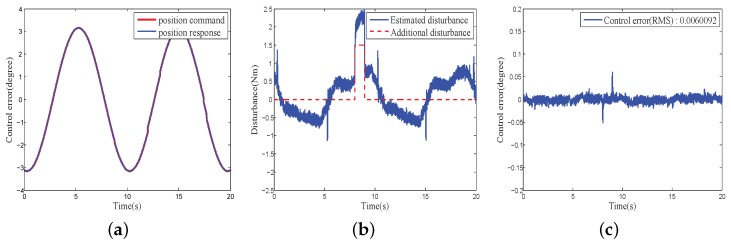
Performance of proposed CHADC controller: (**a**) Control command and response; (**b**) Disturbance estimation; (**c**) Control error.

Figure 16 and Figure 17 show the position responses, disturbance estimation and control error of the COPs under PID + composite disturbance compensator and the proposed CHADC control schemes, respectively. They also present that the control error (RMS) of the proposed CHADC method compared with that of the PID + composite disturbance compensator was reduced from 0.020285 degree to 0.0060092 degree. The maximum fluctuation of the position under the proposed CHADC method was smaller when the same external disturbance load was added, and the position command changed direction.

## 5. Conclusions

In the aerospace field, more and more compact optoelectronic platforms are being applied to unmanned aircraft systems to complete various tasks, such as automatic guidance and search. Aimed at solving the multi-source anti-disturbance problem, a composite hierarchical anti-disturbance controller with phase lag-free multisensor fusion was developed herein. The composite disturbance compensator in the inner layer was combined with a finite-time disturbance observer and internal disturbance modelling, identification and compensation with the MDE algorithm. The pre-identifiable internal disturbance was compensated for by the MDE-based internal disturbance compensator. The external disturbance was evaluated and compensated for by the finite-time disturbance observer. In the meantime, a non-singular terminal sliding mode control was introduced in the outer layer of the proposed CHADC strategy to improve the dynamic response and disturbance attenuation performances. By multisensor fusion, the phase-lead information of a higher-order sensor was adopted, and the filtered information without phase lag was achieved. Stability and performance analyses were conducted. The experiments on a COP were implemented to verify the validity of the proposal. The results highly agreed with the theoretical work and demonstrated that the proposed method achieves a satisfactory multiple disturbance rejection and a robust performance.

## Figures and Tables

**Table 1:** The experiment parameters.

Parameters	Symbols	Values
Nominal mass	$J_{n}$	0.0021667 [kg m^2^]
Nominal damping	$B_{n}$	0.15 [N s/m]
Filter cutoff frequency	$g_{dis}$	50 [rad/s]
Nonlinear parameter of finite-time DOB	α	0.9
Amplitude output limit	$Vout_{max}$	8.5 [V]
Cutoff frequency of sensor filter	*g*	62.8 [rad/s]
Proportional gain of PID	$K_{cp}$	4
Integral gain of PID	$K_{ci}$	8
Derivative gain of PID	$K_{cd}$	0.04
Switching gain of NTSM	*l*	400
Control parameter of NTSM	β	60
Non-singular parameter of NTSM	p/q	5/3

**Table 2:** The parameter of adaptive mutation differential evolution algorithm.

Parameters	Symbols	Values
Number of Decision Variables	$D_{g}$	3
Population Size	Np	30
Crossover Probability	CR	0.9
Basic mutation factor	$F_{0}$	0.5
Maximum number of iterations	$G_{m}$	500
Bound of Scaling Factor 1	$\left[Low_{1},Up_{1}\right]$	[−1,1]
Bound of Scaling Factor 2	$\left[Low_{2},Up_{2}\right]$	[−5,5]
Bound of Scaling Factor 3	$\left[Low_{3},Up_{3}\right]$	[−0.5,0.5]
